# Pegylated Liposomal Doxorubicin (Caelyx^®^) as Adjuvant Treatment in Early-Stage Luminal B-like Breast Cancer: A Feasibility Phase II Trial

**DOI:** 10.3390/curroncol28060433

**Published:** 2021-12-07

**Authors:** Silvia Dellapasqua, Pamela Trillo Aliaga, Elisabetta Munzone, Vincenzo Bagnardi, Eleonora Pagan, Emilia Montagna, Giuseppe Cancello, Raffaella Ghisini, Claudia Sangalli, Mara Negri, Manuelita Mazza, Monica Iorfida, Anna Cardillo, Angela Sciandivasci, Nadia Bianco, Ana Paula De Maio, Monica Milano, Giuseppe Maria Campennì, Loredana Sansonno, Giuseppe Viale, Anna Morra, Maria Cristina Leonardi, Viviana Galimberti, Paolo Veronesi, Marco Colleoni

**Affiliations:** 1 Division of Medical Senology, IEO, European Institute of Oncology IRCCS, 20141 Milan, Italy; Pamela.TrilloAliaga@ieo.it (P.T.A.); elisabetta.munzone@ieo.it (E.M.); emilia.montagna@ieo.it (E.M.); giuseppe.cancello@ieo.it (G.C.); raffaella.ghisini@ieo.it (R.G.); claudia.sangalli@ieo.it (C.S.); mara.negri@ieo.it (M.N.); manuelita.mazza@ieo.it (M.M.); monica.iorfida@ieo.it (M.I.); anna.cardillo@ieo.it (A.C.); angela.sciandivasci@ieo.it (A.S.); nadia.bianco@ieo.it (N.B.); anapaula.demaio@ieo.it (A.P.D.M.); monica.milano@ieo.it (M.M.); giuseppemaria.campenni@ieo.it (G.M.C.); loredana.sansonno@ieo.it (L.S.); marco.colleoni@ieo.it (M.C.); 2 Department of Statistics and Quantitative Methods, University of Milan-Bicocca, 20126 Milan, Italy; vincenzo.bagnardi@ieo.it (V.B.); eleonora.pagan@unimib.it (E.P.); 3 Department of Pathology, European Institute of Oncology IRCCS and University of Milan, 20141 Milan, Italy; giuseppe.viale@ieo.it; 4 Division of Radiotherapy, European Institute of Oncology IRCCS, 20141 Milan, Italy; anna.morra@ieo.it (A.M.); cristina.leonardi@ieo.it (M.C.L.); 5 Division of Senology, IEO, European Institute of Oncology IRCCS, 20141 Milan, Italy; viviana.galimberti@ieo.it (V.G.); paolo.veronesi@ieo.it (P.V.)

**Keywords:** adjuvant chemotherapy, early breast cancer, luminal B-like subtypes, pegylated liposomal doxorubicin (PLD), Caelyx^®^

## Abstract

Background: Adjuvant chemotherapy for Luminal B-like breast cancers usually includes anthracycline-based regimens. However, some patients are reluctant to receive chemotherapy because of side-effects, especially alopecia, and ask for a “less intensive” or personalized approach. Patients and methods: We conducted a phase II feasibility trial to evaluate pegylated liposomal doxorubicin (PLD, Caelyx^®^) as adjuvant chemotherapy. Patients who received surgery for pT1–3, any N, and luminal B-like early-stage breast cancer (EBC) candidates for adjuvant chemotherapy were included. PLD was administered intravenously at 20 mg/m^2^ biweekly for eight courses. Endocrine therapy was given according to menopausal status. Trastuzumab was administered in HER2-positive disease. The primary endpoint was to evaluate the feasibility of this regimen, defined as the ability of a patient to achieve a relative dose intensity (RDI) of at least 85% of the eight cycles of treatment. Secondary endpoints included adverse events (AEs), tolerability, breast cancer-free survival, disease-free survival, and overall survival. Results: From March 2016 to July 2018, 63 patients were included in the trial. Median age was 49 years (range: 33–76), with mostly pre- and peri-menopausal (65%) and stage I–II (94%). Only 5% of patients had HER2-positive EBC. Median RDI was 100% (range: 12.5–100%; interquartile range, IQR: 87.5–100%). The proportion of patients meeting the primary endpoint was 84% (95% confidence interval, CI: 73–92%). Overall, 55 out of 63 enrolled patients completed treatment (87%, 95% CI: 77–94%). Most common AEs were palmar-plantar erythrodysesthesia (12.2%), fatigue (10.4%), and mucositis (8.5%). Only 13% of patients had G3 AEs. None had alopecia. After a median follow-up of 3.9 years (range: 0.3–4.7) two distant events were observed, and all patients were alive at the date of last visit. Conclusions: The trial successfully met its primary endpoint: the regimen was feasible and well tolerated and could be considered for further evaluation as a treatment option for patients with contraindications to standard anthracyclines or requiring a personalized, less intensive approach.

## 1. Introduction

Early-stage breast cancer (EBC) is a potentially curable disease. Surgery remains the cornerstone of the treatment. Adjuvant systemic therapy, such as chemotherapy (CT) and endocrine therapy (ET), is administered depending on stage, grade, biological features of the tumor, and on characteristics and preferences of the patient. Genomic signatures are increasingly driving decisions on whether to offer CT in addition to ET for women with ER-positive, HER2-negative EBC [1]. Luminal B-like breast cancers are associated with a significantly worse prognosis than luminal A-like subtype [2]. Luminal B-like breast cancer can be further classified as HER2-negative (ER positive, HER2-negative, and with high Ki-67/negative or low PgR) or HER2-positive (ER positive, HER2 overexpressed, or amplified, with any Ki-67 and any PgR) [3]. Adjuvant treatment for luminal B-like EBC includes ET for all patients, CT for most patients with HER2-negative disease, and CT with anti-HER2 therapy for HER2-positive disease [4,5].

Anthracyclines are key components of adjuvant chemotherapy regimens for EBC. However, their usefulness is limited by the cumulative dose-dependent cardiotoxicity that may manifest as life-threatening congestive heart failure. To avoid cardiotoxicity, the use of doxorubicin is typically limited to a safe cumulative dose. Liposomal formulations can reduce cardiac risks while maintaining antitumor efficacy [6]. Pegylated liposomal doxorubicin (PLD, Caelyx^®^) is a formulation of doxorubicin in polyethylene-glycol-coated liposomes. PLD is characterized by a very long half-life in the circulation, favorable pharmacokinetic behavior and specific accumulation in tumor tissues [7,8].

PLD is currently approved for the treatment of advanced breast cancer (ABC) as monotherapy in patients who have greater cardiac risks. The efficacy and safety of PLD as single-agent therapy for ABC have been investigated in three phase II trials, with response rates ranging from 18–33%. The most common adverse events (AEs) included stomatitis, skin toxicity, and myelosuppression, while alopecia, cardiotoxicity, nausea, and vomiting were uncommon [9,10,11]. In a case series of 52 patients with ABC, PLD was administered at 20 mg/m^2^ every two weeks. Eight patients (18%) had partial responses (PR) and 17 (39%) stable disease (SD), with a clinical benefit (CB) of 45%. No grade 3–4 AEs were recorded, except for two patients with grade 3 palmar-plantar erythrodysesthesia (PPE). Only two patients developed alopecia [10]. A phase III trial compared the efficacy and safety of PLD with the conventional doxorubicin as first-line therapy in patients with ABC. The median progression-free survival (6.9 vs. 7.8 months; HR = 1.00; 95% CI 0.82–1.22) and overall survival (21 vs. 22 months; HR = 0.94; 95% CI 0.74–1.19) were similar in both treatment groups. Overall, risk of cardiotoxicity was significantly higher with doxorubicin than PLD (HR = 3.16; 95% CI 1.58–6.31; *p* < 0.001). Moreover, the incidences of alopecia, nausea, and vomiting were lower with PLD than conventional doxorubicin [12].

Furthermore, PLD has been shown to be effective and well tolerated as neoadjuvant CT for patients with locally advanced breast cancer [13,14]. Studies that used a combination of PLD with other agents (e.g., taxanes, gemcitabine, cisplatin, and fluorouracil) obtained a clinical response rate that ranged from 71% to 89% [15,16,17,18,19]. In a phase II trial, in 29 patients with locally advanced breast cancer who were not suitable to receive a standard chemotherapy due to age or comorbidities or who asked for a regimen with low incidence of toxic effects irrespective of age, PLD 20 mg/m^2^ biweekly for eight courses was combined with oral metronomic cyclophosphamide 50 mg/day. Eighteen patients (62.1%) achieved a PR (including one pathological complete response), 10 (34.5%) a SD, and one patient experienced a progressive disease [20]. Additionally, a study confirmed that a combined PLD regimen is more effective for patients with HER2-positive breast cancer [21].

Adjuvant anthracyclines and taxanes are associated with a burden of acute and chronic toxicities [22,23,24]. Several patients are reluctant to receive CT because of the fear of its toxic effects and usually ask for a “personalized” approach, even accepting a possible reduction in the treatment efficacy [25]. One of the reasons why patients frequently refuse chemotherapy is the fear of alopecia [26]. Few dermatologic conditions carry as much emotional distress as CT-induced alopecia. Hair loss negatively affects a patient’s perception of appearance, body image, sexuality, and self-esteem [27,28]. Moreover, patients feel deprived of their privacy because the hair loss is readily interpreted by the lay public as associated with having cancer.

We conducted a feasibility phase II trial to evaluate PLD as an adjuvant chemotherapy regimen in patients with early-stage luminal B-like breast cancer.

## 2. Methods

### 2.1. Study Design

We conducted a mono-institutional, single arm, open-label, phase II trial investigating the feasibility of PLD as an adjuvant CT regimen in patients with early-stage luminal B-like breast cancer. The trial was registered in ClinicalTrials.gov (identification code: NCT03712956) and was approved by the local ethics committee and performed in accordance with the Declaration of Helsinki. All patients provided written informed consent before enrollment.

### 2.2. Patients

Eligible patients were women ≥18 years, with Eastern Cooperative Oncology Group performance status (PS) 0–2, who received surgery with no known residual loco-regional disease for pT1–3, any nodal status (TNM 7th edition) breast cancer with either luminal B-like HER2-negative (ER-positive, HER2-negative, and Ki-67 ≥ 20% or PgR-negative or low) or luminal B-like HER2-positive (ER-positive, HER2 over-expressed or amplified, any Ki-67, any PgR) disease. Patients were candidate to adjuvant CT upon investigator’s choice, following decisions taken in the context of the institutional multidisciplinary board, in alignment with local and international clinical guidelines. Patients with synchronous bilateral invasive breast cancer were eligible if all other criteria were met. Further relevant eligibility criteria were no evidence of current infection and no concurrent treatment with other anticancer or investigational agents. Main exclusion criteria were history of any prior ipsi- or contralateral invasive breast cancer or previous or concomitant malignancy diagnosed within the past five years, except for properly treated basal or squamous cell carcinoma of the skin, in-situ carcinoma of the cervix or bladder, or contra- or ipsilateral in-situ breast carcinoma regardless of the date of diagnosis. Furthermore, patients with myocardial infarction within 6 months and with New York Heart Association class III or IV congestive heart failure were excluded.

### 2.3. Treatment

Patients received PLD intravenously at a dose of 20 mg/m^2^ biweekly for eight courses. CT began no later than 8 weeks from surgery. ET comprised ovarian function suppression with LHRH analogues (to be started concurrently with CT) plus tamoxifen 20 mg/day (to be started at the end of CT) for at least 5 years in premenopausal patients or aromatase inhibitors (to be started at the end of CT) for at least 5 years in postmenopausal patients. Trastuzumab was indicated in HER2-positive luminal B-like disease, had to be started at least 4 weeks after the last administration of PLD, and was administered intravenously (loading dose 8 mg/kg, subsequent doses 6 mg/kg) every 3 weeks for 12 months (18 cycles). Radiotherapy was given according to institutional accepted guidelines.

### 2.4. Primary and Secondary Endpoints

The primary endpoint of the study was to evaluate the feasibility of adjuvant PLD for each individual subject. The regimen was considered feasible if the subject was able to achieve a relative dose intensity (RDI) of at least 85% of the 8 treatment cycles. The RDI for each subject was calculated as the ratio between actual dose (AD) and planned dose (PD) (RDI = AD/PD). The PD was calculated based on each subject’s body surface area, and it was the total planned dose for PLD calculated for a full 8-cycle regimen. The AD was the effective dose of PLD for the entire 8-cycle regimen as collected on the relevant reporting form. Secondary endpoints of the trial included AEs (rated according to the Common Terminology Criteria for Adverse Events version 4.0); tolerability (rate of treatment completion); breast cancer-free survival (BCFS, in which events are recurrence of invasive breast cancer at any site, including in-situ recurrences and contralateral disease); disease-free survival (DFS, for which events also include second malignancies and deaths); sites of failure (visceral and non-visceral); and overall survival (OS, for which events are deaths from disease progression or any other cause).

### 2.5. Follow-Up

Patients were followed up after the end of adjuvant treatment. All patients had follow-up visits every six months for the first five years and annually thereafter. In case of relapse, patients were offered standard programs for diagnosis and treatment.

### 2.6. Statistical Analysis

Since the primary endpoint was to determine a patient’s ability to achieve a RDI of at least 85% of the 8 treatment cycles (here defined as success), assuming that a success rate of less than 65% indicates an unfeasible regimen, a two-stage Simon design [29] was used to allow for early termination of the study. The smallest success rate suggesting the regimen was feasible was 80%. In the first phase, 30 patients were recruited. If at least 21 patients had been successfully observed and its tolerability judged adequate by the investigators, another 33 patients would have been enrolled for a total of 63 patients. In the second phase, if 46 or more patients achieve success, the regimen would be considered effective. Therefore, assuming the actual success rate was less than 65%, there was a 64% chance of ending the study early. This two-stage design produced a probability of 90% of concluding that the study was feasible. Type I error was set at 5%.

The variability of the percentage of patients achieving RDI of at least 85% of the 8 treatment cycles was assessed by calculating the exact 95% confidence interval.

Categorical variables were reported with absolute and relative frequencies, continuous variables with median and min-max range, or interquartile (IQR) range.

BCFS, DFS, and OS were assessed using the Kaplan–Meier method.

Sample size calculation was performed using the PASS 2008 software. All other analyses were performed with the SAS software v. 9.4 (SAS Institute, Cary, NC, USA).

## 3. Results

### 3.1. Patients and Tumors’ Characteristics

From March 2016 to July 2018, 63 patients were enrolled. Patients’ and tumors’ characteristics are listed in Table 1. Median age was 49 years (range, 33–76 years). All patients but one had an ECOG PS of 0. Thirty-one patients (49%) were pre-menopausal. The majority of patients (94%) had stage I–II disease. Thirty-three (52%) patients underwent mastectomy, while thirty patients (48%) had conservative breast surgery. Fifty-five patients (87%) had sentinel lymph node biopsy, and eighteen patients (29%) underwent axillary lymph node dissection. Forty-nine patients (77%) received radiotherapy upon completion of CT.

Most patients had pT1c (46%) or pT2 (38%) disease. Thirty-three patients (52%) had negative lymph node status, while 26 patients (41%) had 1–3 involved lymph nodes. Most tumors (90%) showed a ductal histology, mostly grade 2 (27%) and grade 3 (63%). Median Ki-67 labeling index was 33% (range, 20–80%). Median expression of estrogen receptors was 95% (range, 15–100%), and median expression of progesterone receptors was 80% (range, 0–98%). Only 5% of the patients had HER2-positive breast cancer.

### 3.2. Relative Dose Intensity

**Figure 1:**
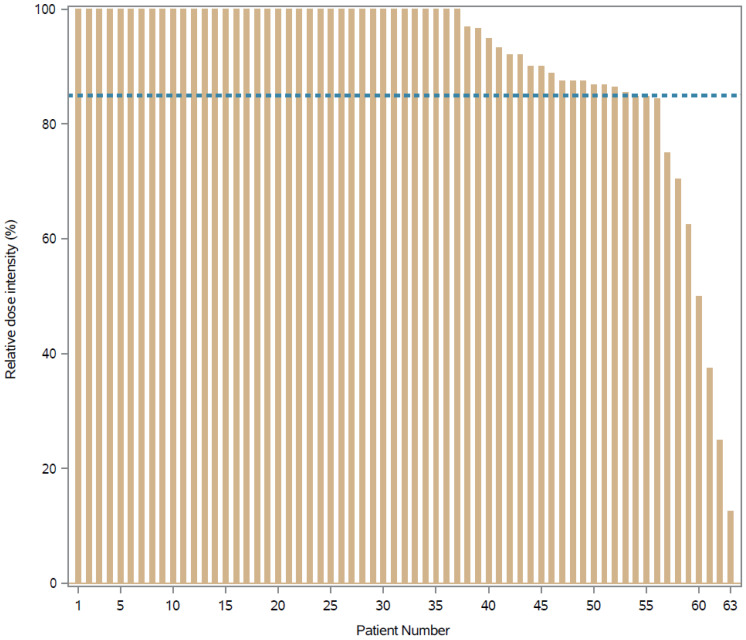
Waterfall plot showing the relative dose intensity (RDI %) by patient (dashed reference line: RDI = 85%).

PLD median planned dose (PD) was 32 mg (range: 25–38; IQR: 30–34), and PLD median actual dose (AD) was 31 mg (range: 0–38; IQR: 29–33). Therefore, PLD median RDI was 100% (range: 12.5–100%; IQR: 87.5–100%). The proportion of patients achieving success was 53/63 = 84% (exact 95% CI: 73–92%). The waterfall plot showing the RDI (%) per patient is shown in Figure 1.

### 3.3. Adverse Events

AEs are listed in Table 2. Overall, 217 AEs were observed, of which 164 (76%) were related to PLD treatment as assessed by investigators. The median number of AEs per patient was 3 (range: 0–11; IQR: 2–5). The distribution of the maximum grade reported per patient was G0 in three cases (5%); G1 in 20 cases (32%); G2 in 32 cases (51%); and G3 in eight cases (13%). None of the patients experienced grade 4–5 toxicity or had serious AEs. Most common related AEs included skin toxicities (35.9%), mainly PPE (12.2%), fatigue (10.4%), mucositis (8.5%), and nausea (7.3%), while liver toxicity (2.4%) and neutropenia (0.6%) were rare. None of the patients developed alopecia. One patient (0.6%) had a mild-moderate asymptomatic reduction in left ventricular ejection fraction (LVEF 45–50%) after the third cycle. She had been previously exposed to anthracycline-based chemotherapy in her youth for osteosarcoma of the right lower limb from which she subsequently recovered.

### 3.4. Tolerability

Tolerability was defined as the rate of treatment completion. Fifty-five out of 63 enrolled patients completed the adjuvant program (87%, 95% CI: 77–94%). Eight patients (12.7%) discontinued treatment, seven due to toxicity and one due to post-surgical complications. Of the 26 patients who did not achieve 100% of RDI, eight patients (12.7%) had a treatment delay followed by resumption of PLD at a reduced dose, one patient (1.6%) had a treatment delay followed by resumption of PLD at a reduced dose followed by another treatment delay, six patients (9.5%) had to reduce PLD dose, two patients (3.2%) had to reduce PLD dose then had another treatment delay, one patient (1.6%) had to reduce PLD dose then permanently discontinued PLD, six patients (9.5%) permanently discontinued PLD, one patient (1.6%) had to stop PLD infusion and later permanently discontinued PLD, and one patient (1.6%) had a treatment delay. Considering all the 63 enrolled patients, the median number of delayed days was 28 (IQR 21–33; min-max range 2–59). The most common cause of treatment discontinuation was skin toxicity (6%). Four patients did not receive the last cycle of PLD: two had G3 skin rash, one had hyperbilirubinemia, and one had post-surgery complications. Details on tolerability are reported in Table 3.

### 3.5. Survival

**Figure 2:**
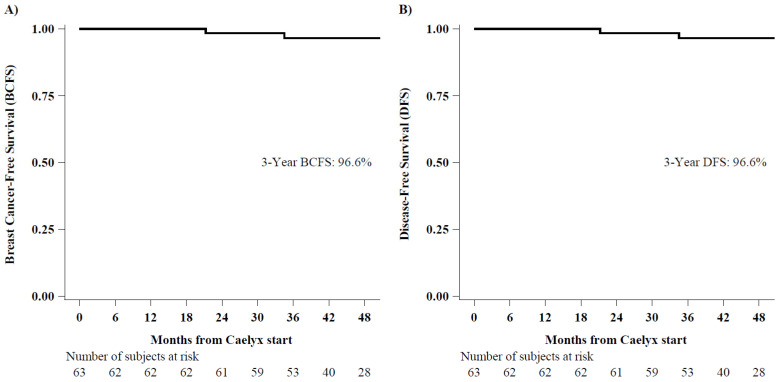
Breast Cancer-Free Survival (panel **A**) and Disease-Free Survival (panel **B**).

Secondary endpoints of the study included BCFS, DFS and OS. The median follow-up was 3.9 years (IQR 3.3–4.3 years, min-max 0.3–4.7 years) as of 15 February 2021. During the follow-up period, two distant events were observed: bone-only recurrence after 21 months in one patient and liver and lung metastases at 34 months in another patient. Neither second primary malignancies or deaths were observed, leading to identical Kaplan–Meier DFS and BCFS curves (Figure 2A,B). The three-year BCFS and DFS was 96.6%. All patients were alive at the date of the last follow-up visit.

## 4. Discussion

This phase II trial was conducted to assess the feasibility of PLD administered intravenously at 20 mg/m^2^ biweekly for eight courses as adjuvant therapy in patients with luminal B-like EBC. Overall, based on the pre-specified criteria that the regimen would be considered feasible if ≥80% of evaluable patients were able to achieve an RDI of ≥85%, the treatment regimen was deemed feasible. The feasibility rate was 84% (95% CI: 73–92%), and the median RDI was 100% (IQR: 87.5–100%). These data demonstrate that eight cycles of PLD were generally well tolerated in the adjuvant setting and support the conduct of larger comparative studies to evaluate the efficacy of this regimen.

RDI is an important prognostic factor reflecting the degree of adherence, safety, and tolerability of treatment. Maintaining the RDI above an optimal trough of 85% has been shown to correlate with increased rates of DFS and OS [30,31]. In this study, we achieved a three-year DFS of 96.6%, and all patients were alive at the last follow-up date. However, these survival data are still immature, and longer follow-up is required for more consistent data. A case-control study comparing PLD-based and conventional anthracycline-based regimens in 102 women with stage I–IIIa breast cancer (63.7% and 71.6% of patients in the two treatment groups had hormone receptor positive disease, respectively) found that five-year DFS was comparable for PLD and epirubicin (81.3% vs. 82.3% respectively; *p* = 0.939), but there was higher grade 3 and 4 toxicity in patients receiving epirubicin-based regimens than in those receiving PLD-based regimens except for hand-foot syndrome [32].

In this trial, PLD had a favourable safety profile, comparable to the one previously reported in other studies [15,16,17,18,19,20,21]. In particular, in a phase II preoperative trial, treatment with PLD 20 mg/m^2^ given biweekly for eight courses in combination with metronomic cyclophosphamide 50 mg/day orally was well tolerated, with no grade 4 toxicities and with grade 3 skin toxicity in three patients and hand-foot syndrome in four patients [20]. Most common AEs in the present study were skin toxicity (particularly palmar-plantar erythrodysesthesia), fatigue, and mucositis; conversely, nausea, hematological toxicity, and liver toxicity were rare. None of the patients experienced alopecia. The cardiac safety of PLD was confirmed in this study. Only one patient (0.6%), previously exposed to anthracyclines, experienced an asymptomatic decrease in LVEF of ≥10%. Recent prospective studies report frequent subclinical left ventricular dysfunction (defined as an absolute decrease in LVEF of >10%) in 10% to 50% of patients receiving anthracyline-based therapy [33]. In this study, we also treated three patients (5%) with HER2-positive breast cancer with PLD followed by trastuzumab for one year, and these patients did not experience cardiotoxicity. The use of PLD in combination with trastuzumab for HER2-positive breast cancer could offer an effective anthracycline-based regimen without the known cardiotoxic morbidity associated with conventional anthracyclines plus trastuzumab [34]. This hypothesis should be further investigated in future clinical trials in the neo-adjuvant setting.

A potential limitation of this study resides in its design since this was a mono-institutional, single-arm, non-comparative study. Another possible limitation is the relatively immature follow up: since breast cancer recurrences can occur well after five years, longer follow-up is needed to evaluate long term outcome and safety profile of this treatment. Furthermore, results of our trial should be viewed in the context of currently used genomic signatures, which were not available at the time the trial was conducted, whose adoption in clinical practice has dramatically lowered the use of adjuvant CT without adversely affecting clinical outcomes. As for the strengths of this trial, we should consider that this was a well-conducted, prospective phase II trial; adjuvant treatment was discussed and agreed upon at a multidisciplinary meeting in a comprehensive cancer center; and treatments were all delivered in a single institution so that variability in the treatment patterns of non-systemic treatments was consistent.

In conclusion, our study successfully met its primary endpoint: the adjuvant regimen with PLD biweekly for eight administrations was feasible and well tolerated. This study is hypothesis generating and supports the conduct of larger comparative trials to assess whether adjuvant chemotherapy with PLD could be an option for selected early-stage luminal B-like breast cancer, particularly for patients with contraindications to traditional anthracycline-containing regimens, for those requiring a less intensive and personalized approach, and finally for those patients who would refuse chemotherapy to avoid the risk of alopecia.

## Figures and Tables

**Table 1:** Patients and Tumors’ Characteristics.

Variable	Level	*N* = 63	Percentage (%)
Age (years)	Median (min–max)	49 (33–76)	
Sex	Female	63	100.0
Menopausal status	Pre	31	49.2
	Peri	10	15.9
	Post	22	34.9
ECOG PS	0	62	98.4
	1	1	1.6
Type of surgery	Mastectomy	33	52.4
	Conservative	30	47.6
Histological type	Ductal	57	90.4
	Lobular	2	3.2
	Mixed	2	3.2
	Other	2	3.2
SNB	No	8	12.7
	Yes	55	87.3
ALND	No	45	71.4
	Yes	18	28.6
Tumor size (mm)	Median (min–max)	18 (5–65)	
T-stage	pT1a	1	1.6
	pT1b	6	9.5
	pT1c	29	46.0
	pT2	24	38.1
	pT3	3	4.8
N-stage	pN0/NSentNeg	33	52.4
	pN1mi	7	11.1
	pN1a	19	30.2
	pN2a	2	3.2
	pN3a	2	3.2
TNM stage	IA	23	36.5
	IB	3	4.8
	IIA	18	28.6
	IIB	13	20.6
	IIIA	4	6.3
	IIIC	2	3.2
Grading	G2	17	27.0
	G3	46	73.0
Ki-67 expression	Median expression (min–max)	33% (20–80%)	
	≤20%	2	3.2
	>20%	61	96.8
HER2 status	Negative	60	95.2
	Positive	3	4.8
ER expression	Median expression (min–max)	95% (15–100%)	
	>1%	63	100.0
	≤1%	0	0
PgR expression	Median expression (min–max)	80% (0–98%)	
	>1%	53	84.1
	≤1%	10	15.9

ECOG PS, Eastern Cooperative Oncology Group Scale Performance Status; SNB, sentinel lymph node biopsy; ALND, axillary lymph node dissection.

**Table 2:** Adverse Events *.

System Preferred Term	All Grades *N* (col%)	Grade 1 *n* (col%)	Grade 2 *n* (col%)	Grade 3 *n* (col%)	Grade 4–5 *n* (col%)
All related AEs	164	108	49	7	0
Skin and appendages	71 (43.3)	45 (63.4)	21 (29.6)	5 (7.0)	0
Palmar-plantar erythrodyestesia	20 (12.2)	12 (11.1)	8 (16.3)	0	0
Erythema	13 (7.9)	5 (4.6)	4 (8.2)	4 (57.1)	0
Rash	10 (6.1)	7 (6.5)	3 (6.1)	0	0
Folliculitis	10 (6.1)	8 (7.4)	2 (4.1)	0	0
Eczema	2 (1.2)	2 (1.9)	0	0	0
Skin dyschromia	2 (1.2)	2 (1.9)	0	0	0
Dry skin	2 (1.2)	2 (1.9)	0	0	0
Onychopathy	3 (1.8)	1 (0.9)	2 (4.1)	0	0
Itch	5 (3.1)	4 (3.7)	1 (2.0)	0	0
Other	4 (2.4)	2 (1.9)	1 (2.0)	1 (14.3)	0
Gastrointestinal	42 (25.6)	31 (73.8)	11 (26.2)	0	0
Mucositis	14 (8.5)	11 (10.2)	3 (6.1)	0	0
Nausea	12 (7.3)	11 (10.2)	1 (2.0)	0	0
Epigastralgia	3 (1.8)	2 (1.9)	1 (2.0)	0	0
Constipation	8 (4.9)	3 (2.8)	5 (10.2)	0	0
Diarrhea	1 (0.6)	1 (0.9)	0	0	0
Dysgeusia	1 (0.6)	1 (0.9)	0	0	0
Abdominal pain	1 (0.6)	1 (0.9)	0	0	0
Other	2 (1.2)	1 (0.9)	1 (2.0)	0	0
Hepatic	4 (2.4)	1 (25.0)	3 (75.0)	0	0
Transaminases increased	3 (1.8)	1 (0.9)	2 (4.1)	0	0
Bilirubin increased	1 (0.6)	0	1 (2.0)	0	0
Systemic	27 (16.5)	22 (81.5)	4 (14.8)	1 (3.7)	0
Fatigue	17 (10.4)	14 (13.0)	3 (6.1)	0	0
General malaise	3 (1.8)	2 (1.9)	0	1 (14.3)	0
Sweats	1 (0.6)	1 (0.9)	0	0	0
Fever	1 (0.6)	0	1 (2.0)	0	0
Headache	2 (1.2)	2 (1.9)	0	0	0
Dizziness	1 (0.6)	1 (0.9)	0	0	0
Hot flashes	2 (1.2)	2 (1.9)	0	0	0
Infusion Related	4 (2.4)	0	4 (100.0)	0	0
Infusion reaction	1 (0.6)	0	1 (2.0)	0	0
Chest tightness	2 (1.2)	0	2 (4.1)	0	0
Lower back pain	1 (0.6)	0	1 (2.0)	0	0
Hematopoietic	2 (1.2)	0	1 (50.0)	1 (50.0)	0
Leukopenia	1 (0.6)	0	1 (2.0)	0	0
Neutropenia	1 (0.6)	0	0	1 (14.3)	0
Cardiovascular	2 (1.2)	1 (50.0)	1 (50.0)	0	0
Hypotension	1 (0.6)	1 (0.9)	0	0	0
Decreasead LVEF	1 (0.6)	0	1 (2.0)	0	0
Neuromuscular	9 (5.5)	6 (66.7)	3 (33.3)	0	0
Arthromyalgia	2 (1.2)	2 (1.9)	0	0	0
Paraesthesia	3 (1.8)	2 (1.9)	1 (2.0)	0	0
Dysesthesia	3 (1.8)	1 (0.9)	2 (4.1)	0	0
Neuralgia	1 (0.6)	1 (0.9)	0	0	0
Urogenital	3 (1.8)	2 (66.7)	1 (33.3)	0	0
Vaginal dryness	1 (0.6)	0	1	0	0
Cystitis	2 (1.2)	2 (1.9)	0	0	0

col%, column percentages; LVEF, left ventricular ejection fraction. * All percentages refer to the total number of related AEs (overall and according to grade).

**Table 3:** Treatment Tolerability.

All Included Cases		*N* = 63	Percentage(%)
	Treatment completed per protocol	55	87.3
	Treatment interrupted	8	12.7
Cause of definitive interruption	HFS	2	3.2
	Rash	2	3.2
	Hyperbilirubinemia	1	1.6
	Decrease ejection fraction	1	1.6
	Infusion reaction	1	1.6
	Physician decision	1	1.6
Relative dose intensity			
	85% achieved	55	87.3
	85% not achieved	8	12.7

## Data Availability

The data presented in this study are available on request from the corresponding author. The data are not publicly available due to privacy restrictions.
